# Three-dimensional Anthropometric Analysis of Racial and Ethnic Differences in Unilateral and Bilateral Cleft Nasal Deformity

**DOI:** 10.1177/10556656231167066

**Published:** 2023-03-27

**Authors:** Lucas M. Harrison, Naomi A. Cole, Christopher A. Derderian, Rami R. Hallac

**Affiliations:** 1 Department of Plastic Surgery, University of Texas Southwestern Medical Center, Dallas, TX, USA; 2 Analytical Imaging and Modeling Center, Children's Medical Center, Dallas, TX, USA

**Keywords:** cleft lip and palate, anthropometry, nasal morphology

## Abstract

**Objective:**

This study used three-dimensional measurements to provide a nasolabial analysis of patients with unilateral cleft lip and palate (UCLP), bilateral cleft lip and palate (BCLP), and controls across different races and ethnicities.

**Design:**

A retrospective comparative study.

**Setting:**

Tertiary care pediatric institution.

**Patients, Participants:**

The study included 90 patients with UCLP, 43 patients with BCLP, and 90 matched controls. Patients are separated as self-identified Caucasian, Hispanic, or African American.

**Main Outcome Measure(s):**

Nasal length, nasal protrusion, columellar height, columellar width, tip width, alar width, alar base width, nasolabial angle, upper lip length, philtrum length, nostril height, and nostril width.

**Results:**

All UCLP groups had significantly greater columella and tip widths and decreased nasolabial angles than controls. All BCLP groups had significantly greater columella width, tip width, nasolabial angle, and nostril widths. Upper lip length, philtrum length, and nostril height were significantly decreased in BCLP compared to controls. Across UCLP groups, African Americans had significantly decreased nasal protrusion and columella height and a significantly increased columella width compared to Caucasians and Hispanics. Alar and alar base widths were significantly different between all groups. Across BCLP groups, the Caucasian nostril width was significantly less than the African Americans.

**Conclusions:**

These findings suggest that when correcting nasolabial characteristics in patients with cleft lip, it is important to consider racial and ethnic differences to achieve a normal appearance. Specifically, goals for alar width, alar base width, nasal tip, and projection should be tailored to the patient's race and ethnicity.

## Introduction

The complex nasal deformity associated with complete unilateral and bilateral cleft lip and palate has been well documented in the literature. In individuals with complete unilateral cleft lip and palate (UCLP), the cleft-side lower lateral cartilage is inferiorly and posterolaterally displaced compared to the non-cleft side.^1,2^ Alar cartilage displacement coincides with an asymmetric nasal tip, deviated columella, and a deviated caudal septum.^1,2^ In the bilateral cleft lip and palate (BCLP) nasal deformity, the lateral crura are flattened and elongated. In contrast, the medial crura are shortened and widely separated at the nasal tip.^3^ These changes give rise to a shortened columella, laterally displaced alae, with a flattened, bifid-appearing nasal tip.^3^

Patients with cleft lip and palate usually undergo a secondary rhinoplasty at dentoskeletal maturity. Reconstructive surgeons should understand nasal morphology across various racial and ethnic groups to perform a congruent secondary rhinoplasty in patients with cleft nasal deformity. Anthropometric indices such as nasal width and height vary significantly among different racial and ethnic groups, including Caucasian, Asian, Middle Eastern, and African American.^4^ These variations in nasal anatomy have been extensively documented for use in adult aesthetic rhinoplasty planning.^5–9^

Normative data on nasal morphology for each racial and ethnic group can be used in surgical planning to achieve a congruent outcome from secondary rhinoplasty. Likewise, pre-operative anthropometric data on the specific characteristics associated with UCLP and BCLP nasal deformity can provide an objective and consistent foundation for operative planning. Understanding the differences in the nasal indices of the cleft populations and their matched peers at dentoskeletal maturity can better inform surgeons which areas to prioritize during secondary rhinoplasty. The current literature has a variety of anthropometric analyses on cleft nasal deformity pre-dentoskeletal maturity or post-secondary rhinoplasty. However, there are no anthropometric analyses on the racial and ethnic variation in patients with UCLP and BCLP cleft nasal deformity at dentoskeletal maturity, pre-secondary rhinoplasty.

This purpose of this study is to provide a comprehensive nasolabial analysis of patients with cleft nasal deformity before secondary rhinoplasty across racial and ethnic groups. The study aims to gather normative data on nasal morphology for each group to be used in surgical planning to achieve a racial and ethnic congruent outcome from cleft rhinoplasty. Additionally, the study aims to gather pre-operative anthropometric data on the specific characteristics associated with UCLP and BCLP to provide an objective and consistent foundation for operative planning. By understanding the differences in the nasal indices of cleft populations and their age-matched peers at dentoskeletal maturity, the study aims to better inform surgeons which areas to prioritize during secondary rhinoplasty.

## Methods

All patients included in the study were diagnosed with UCLP or BCLP and underwent primary cleft and nasal reconstruction at Children's Health Medical Center in Dallas. After obtaining IRB approval from the University of Texas Southwestern Medical Center, we retrospectively analyzed the three-dimensional images of 90 patients with UCLP and 43 patients with BCLP before orthognathic or secondary cleft rhinoplasty. The patients were classified as Caucasian, Hispanic, or African American based on self-reported identifiers. Additionally, we analyzed 90 control patients who were age, sex, race, and ethnicity matched. No patients with syndromic cleft lip and palate or other congenital or acquired craniofacial malformation were included. Patient characteristics are shown in Table 1.

**Table 1.** Patient Characteristics.

	Total	Left Side	Right Side	Males	Females	Average Age (Mean ± STD)
*Caucasian*						
UCLP Patients	21	10	11	11	10	15.86 ± 0.74
BCLP Patients	6	-	-	5	1	15.46 ± 1.35
Control Patients	21	-	-	11	10	15.78 ± 1.00
*Hispanic*						
UCLP Patients	62	38	24	44	18	16.33 ± 1.04
BCLP Patients	30	-	-	14	16	15.90 ± 1.00
Control Patients	62	-	-	44	18	15.67 ± 1.44
*African American*						
UCLP Patients	7	4	3	4	3	16.81 ± 1.15
BCLP Patients	7	-	-	3	4	15.59 ± 1.28
Control Patients	7	-	-	4	3	16.72 ± 0.97

### Three-Dimensional Photography

**Figure 1.**
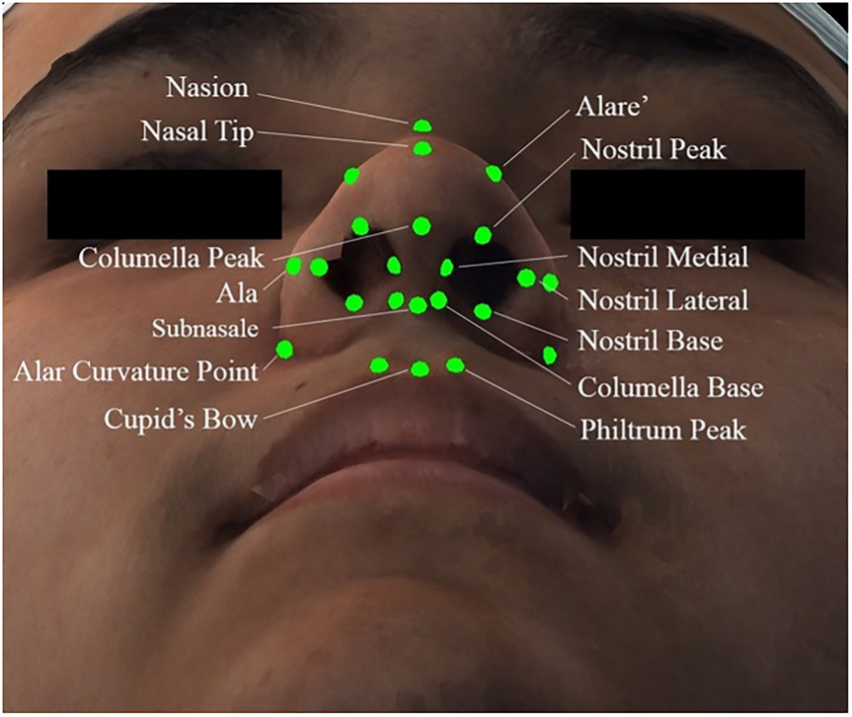
Nasal and upper lip landmarks.

Three-dimensional facial photographs were acquired using a 3dMD imaging system (3dMD, Atlanta, GA). A thin nylon stockinette was placed on each patient's head, and all images were capture with a neutral head posture and expression. Images were collected by our trained laboratory staff. Virtual 3D-derived models were analyzed using 3dMD Vultus software (3dMD, Atlanta, GA). Landmarks placed included the nasion, nasal tip, subnasale, columella peak, columella base, cupid's bow trough, philtrum peak, nostril peak, nostril base, nostril medial, nostril lateral, ala, alare’, and alar curvature point (Figure 1).

Twelve measurements were collected from each patient. They included nasal length (distance from nasion to nasal tip), nasal protrusion (distance from subnasale to nasal tip), columella height (distance from subnasale to columella peak), columella width (distance between the medial nostril points), tip width (distance between the alare’ points), alar width (distance between ala points), alar base width (distance between alar curvature points), upper lip length (distance from subnasale to cupid's bow trough), nasolabial angle (angle from columella peak to subnasale to cupid's bow trough), philtrum length (distance from columella base to philtrum peak bilaterally), nostril height (distance from nostril base to peak bilaterally), and nostril width (distance from the nostril medial to lateral bilaterally).

### Statistical Analysis

All measurements were assessed for inter-observer and intra-observer reliability by Pearson's Correlation Coefficient analysis. Two observers completed each measurement two times. The average value and standard deviation were calculated for each group. Two-sample unpaired t-tests were used to compare UCLP and BCLP patients against matched controls in each race and ethnicity group and comparison between UCLP and BCLP groups. Statistical significance was considered a value of *P* < .05.

## Results

Pearson's Correlation Coefficient inter-rater and intra-rater reliability showed a strong correlation with r = 0.98 and r = 0.99, respectively. The results of the Caucasian group analysis are shown in Table 2. The columella width, tip width, alar width, and alar base width were more significant in the UCLP and BCLP groups than controls. The control group had a significantly greater nasolabial angle than the UCLP patients and a significantly smaller angle than the BCLP patients. Compared to controls, the upper lip measurements in the BCLP group showed significantly shorter upper lip and philtrum lengths. The nostrils in BCLP patients had a significantly wider width and a shorter height than controls.

**Table 2.** Caucasian UCLP, BCLP, and Control Results (Mean mm/° ± STD).

	UCLP	BCLP	Control	*P* Value		
				U vs C	A vs Un	B vs C
Nasal Length	42.84 ± 4.51	44.67 ± 4.33	42.22 ± 2.84	.598	-	.110
Nasal Protrusion	20.18 ± 1.93	19.57 ± 2.37	19.88 ± 1.67	.593	-	.722
Columella Height	10.57 ± 1.45	9.06 ± 2.37	9.89 ± 0.92	.075	-	.121
Columella Width	8.48 ± 1.07	9.09 ± 1.45	7.16 ± 1.03	**<**.001*	-	**<**.001*
Tip Width	21.06 ± 2.51	24.19 ± 1.83	17.69 ± 1.58	**<**.001*	-	**<**.001*
Alar Width	33.66 ± 3.12	37.66 ± 0.77	31.21 ± 2.54	.008*	-	**<**.001*
Alar Base Width	33.66 ± 3.51	38.06 ± 0.65	31.33 ± 2.18	.013*	-	**<**.001*
Upper Lip Length	11.72 ± 1.46	10.19 ± 1.07	12.55 ± 2.60	.209	-	.042*
Nasolabial Angle	106.65° ± 9.64°	121.93° ± 12.74°	112.90° ± 8.25°	.029*	-	.047*
Affected Philtrum Length	11.78 ± 1.92	10.11 ± 0.81	12.04 ± 2.19	.867	.886	.005*
Unaffected Philtrum Length	11.94 ± 2.02			.653		
Affected Nostril Height	10.88 ± 1.79	10.55 ± 1.71	11.52 ± 1.30	.109	.411	.038*
Unaffected Nostril Height	11.33 ± 1.76			.632		
Affected Nostril Width	10.80 ± 1.51	12.54 ± 1.68	10.34 ± 1.01	.154	.997	**<**.001*
Unaffected Nostril Width	10.81 ± 1.36			.132		

Abbreviations: U, UCLP; B, BCLP; C, Control; A, Affected; Un, Unaffected.

Asterisk (*) represents statistical significance between groups (*P* < .05). U (UCLP), B (BCLP), C (Control), A (Affected), Un (Unaffected).

The Hispanic group results are shown in Table 3. The UCLP and BCLP groups had significantly greater columella width, tip width, alar width, and alar base width than the control group. The UCLP group had a significantly smaller nasolabial angle than the controls, while the BCLP group had a significantly larger angle than the control group. The upper lip and philtrum lengths in the BCLP group were significantly shorter than in the controls. The BCLP group had a significantly wider nostril width and significantly shorter nostril height than the control group.

**Table 3.** Hispanic UCLP, BCLP, and Control Measurement Results (Mean mm/°  STD).

	UCLP	BCLP	Control	*P* Value		
				U vs C	A vs Un	B vs C
Nasal Length	44.01 ± 4.27	41.70 ± 3.83	43.10 ± 4.12	.229	-	.122
Nasal Protrusion	21.14 ± 2.12	19.80 ± 3.55	20.27 ± 2.84	.056	-	.491
Columella Height	10.83 ± 1.96	9.20 ± 3.01	10.13 ± 2.16	.060	-	.094
Columella Width	8.26 ± 1.51	8.74 ± 2.22	7.46 ± 1.55	.004*	-	.002*
Tip Width	20.81 ± 2.51	22.92 ± 2.64	16.43 ± 3.00	**<**.001*	-	**<**.001*
Alar Width	37.52 ± 3.46	38.75 ± 3.79	35.00 ± 3.20	**<**.001*	-	**<**.001*
Alar Base Width	36.77 ± 2.81	38.73 ± 3.35	35.13 ± 3.08	.002*	-	**<**.001*
Upper Lip Length	11.91 ± 4.86	10.64 ± 1.88	12.21 ± 2.09	.657	-	**<**.001*
Nasolabial Angle	100.56° ± 12.13°	125.89° ± 16.77°	112.98° ± 10.61°	**<**.001*	-	**<**.001*
Affected Philtrum Length	12.17 ± 2.33	10.69 ± 2.75	12.32 ± 2.21	.677	.990	**<**.001*
Unaffected Philtrum Length	12.18 ± 2.14			.675		
Affected Nostril Height	11.03 ± 2.65	10.45 ± 2.94	11.66 ± 2.24	.088	.656	.002*
Unaffected Nostril Height	11.22 ± 2.14			.198		
Affected Nostril Width	12.04 ± 2.28	13.23 ± 2.13	11.92 ± 2.13	.711	.539	**<**.001*
Unaffected Nostril Width	11.76 ± 2.79			.673		

Abbreviations: U, UCLP; B, BCLP; C, Control; A, Affected; Un, Unaffected.

Asterisk (*) represents statistical significance between groups (*P* < .05).

The African American group results are shown in Table 4. The columella width and tip width were significantly wider in the UCLP and BCLP groups compared to the controls. The upper lip length and philtrum lengths were significantly shorter in BCLP patients compared to the control group. The control group had a significantly greater nasolabial angle than the UCLP group and was significantly smaller than the BCLP group.

**Table 4.** African American UCLP, BCLP, and Control Results (Mean mm/°  STD).

	UCLP	BCLP	Control	*P* Value		
				U vs C	A vs Un	B vs C
Nasal Length	41.51 ± 3.22	42.58 ± 3.94	42.10 ± 1.90	.681	-	.778
Nasal Protrusion	17.84 ± 1.38	17.65 ± 0.31	17.82 ± 0.64	.969	-	.539
Columella Height	8.96 ± 0.85	8.26 ± 1.96	8.40 ± 1.52	.408	-	.891
Columella Width	9.56 ± 1.58	10.24 ± 2.41	6.63 ± 0.71	**<**.001*	-	.003*
Tip Width	22.28 ± 2.19	23.54 ± 3.04	18.75 ± 2.47	.015*	-	.007*
Alar Width	41.63 ± 3.43	40.88 ± 3.92	39.17 ± 3.35	.199	-	.397
Alar Base Width	41.93 ± 3.78	40.56 ± 3.97	39.21 ± 0.90	.089	-	.399
Upper Lip Length	12.82 ± 1.13	11.16 ± 0.52	13.22 ± 2.15	.815	-	.030*
Nasolabial Angle	100.19° ± 9.16°	124.03° ± 22.11°	111.97° ± 8.90°	.031*	-	.042*
Affected Philtrum Length	12.95 ± 1.43	11.25 ± 1.77	13.23 ± 0.99	.905	.835	.001*
Unaffected Philtrum Length	13.08 ± 0.80			.885		
Affected Nostril Height	11.06 ± 2.40	9.84 ± 3.14	11.44 ± 1.73	.679	.588	.107
Unaffected Nostril Height	10.49 ± 1.20			.211		
Affected Nostril Width	13.96 ± 2.43	14.10 ± 1.62	14.00 ± 1.45	.962	.932	.867
Unaffected Nostril Width	13.85 ± 2.01			.851		

Abbreviations: U, UCLP; B, BCLP; C, Control; A, Affected; Un, Unaffected.

Asterisk (*) represents statistical significance between groups (*P* < .05).

The results of the UCLP comparison between racial and ethnic groups are shown in Table 5. The African American group had significantly less nasal protrusion and columella height and significantly greater columella width than the Caucasian and Hispanic groups. The alar width, alar base width, and affected nostril width were more significant in the Hispanic group than in the Caucasian group. The same measurements in the African American group were more significant than in the Caucasian and Hispanic groups. The unaffected nostril width was significantly larger in African Americans than in the Caucasian group.

**Table 5.** Unilateral Cleft lip and Palate Measurement Results (Mean mm/ STD).

	Caucasian	Hispanic	African American	*P* Value		
				C vs H	H vs AA	C vs AA
Nasal Length	42.84 ± 4.51	44.01 ± 4.27	41.51 ± 3.22	.286	.138	.479
Nasal Protrusion	20.18 ± 1.93	21.14 ± 2.12	17.84 ± 1.38	.069	**<**.001*	.007*
Columella Height	10.57 ± 1.45	10.83 ± 1.96	8.96 ± 0.85	.584	.016*	.010*
Columella Width	8.48 ± 1.07	8.26 ± 1.51	9.56 ± 1.58	.536	.034*	.048*
Tip Width	21.06 ± 2.51	20.81 ± 2.51	22.28 ± 2.19	.690	.141	.263
Alar Width	33.66 ± 3.12	37.52 ± 3.46	41.63 ± 3.43	**<**.001*	.004*	**<**.001*
Alar Base Width	33.66 ± 3.51	36.77 ± 2.81	41.93 ± 3.78	**<**.001*	**<**.001*	**<**.001*
Upper Lip Length	11.72 ± 1.46	11.91 ± 4.86	12.82 ± 1.13	.857	.627	.082
Nasolabial Angle	106.65° ± 9.64°	100.56° ± 12.13°	100.19° ± 9.16°	.140	.937	.133
Affected Philtrum Length	11.78 ± 1.92	12.18 ± 2.13	12.95 ± 1.43	.455	.356	.153
Unaffected Philtrum Length	11.94 ± 2.02	12.17 ± 2.33	13.08 ± 0.80	.685	.311	.160
Affected Nostril Height	10.88 ± 1.79	11.03 ± 2.65	11.06 ± 2.40	.813	.975	.833
Unaffected Nostril Height	11.33 ± 1.76	11.22 ± 2.14	10.49 ± 1.20	.827	.383	.253
Affected Nostril Width	10.80 ± 1.51	12.04 ± 2.28	13.96 ± 2.43	.023*	.40*	**<**.001*
Unaffected Nostril Width	10.81 ± 1.36	11.76 ± 2.79	13.85 ± 2.01	.136	.059	**<**.001*

Abbreviations: C, Caucasian; H, Hispanic; AA, African American.

Asterisk (*) represents statistical significance between groups (*P* < .05).

The BCLP comparison results between racial and ethnic groups are shown in Table 6. The nostril width in the African American group was significantly wider than in the Caucasian group. All other measurements were not significantly different between Caucasian, Hispanic, and African American BCLP groups.

**Table 6.** Bilateral Cleft lip and Palate Measurement Results (Mean mm/°  STD).

	Caucasian	Hispanic	African American	*P* Value		
				C vs H	H vs AA	C vs AA
Nasal Length	44.67 ± 4.33	41.70 ± 3.83	42.58 ± 3.94	.098	.59	.381
Nasal Protrusion	19.57 ± 2.37	19.80 ± 3.55	17.65 ± 3.07	.885	.123	.055
Columella Height	9.06 ± 1.62	9.20 ± 3.01	8.27 ± 1.96	.918	.445	.449
Columella Width	9.09 ± 1.45	8.74 ± 2.22	10.24 ± 2.41	.714	.123	.333
Tip Width	24.19 ± 1.83	22.92 ± 2.64	23.54 ± 3.04	.272	.592	.657
Alar Width	37.66 ± 0.77	38.75 ± 3.79	40.88 ± 3.92	.494	.192	.075
Alar Base Width	38.06 ± 0.65	38.73 ± 3.35	40.56 ± 3.97	.631	.219	.159
Upper Lip Length	10.19 ± 1.07	10.64 ± 1.88	11.16 ± 0.52	.574	.477	.055
Nasolabial Angle	121.93° ± 12.74°	125.89° ± 16.77°	130.60° ± 19.72°	.590	.521	.377
Philtrum Length	10.11 ± 0.81	10.69 ± 2.30	11.25 ± 1.77	.402	.394	.052
Nostril Height	10.55 ± 1.71	10.45 ± 2.94	9.84 ± 3.14	.913	.493	.494
Nostril Width	12.54 ± 1.68	13.23 ± 2.13	14.10 ± 1.62	.292	.159	.024*

Abbreviations: C, Caucasian; H, Hispanic; AA, African American.

Asterisk (*) represents statistical significance between groups (*P* < .05).

## Discussion

In the United States, the census predicts that the Hispanic population will increase by 56% from 62.3 to 111.2 million, and the African American population will increase from 44.7 to 60.6 million citizens by 2060.^10^ As a result, there will likely be a corresponding increase in minority patients seeking treatment at cleft lip and palate centers throughout the country. Therefore, it is crucial for surgeons to understand the racial and ethnic differences in nasolabial anatomy in addition to the patient's esthetic desires for ideal surgical planning for secondary cleft rhinoplasty. Previous studies on nasolabial measurements at skeletal maturity in UCLP and BCLP patients have only included Caucasian or Asian patients.^11–16^ This study examined nasolabial anthropometrics before secondary cleft rhinoplasty across multiple racial and ethnic groups.

It is essential to understand the underlying anatomic characteristics and deformities caused by cleft lip and palate in different racial and ethnic groups to develop a surgical plan that corrects the deformity and produces an esthetically pleasing result. Racial and ethnic nasolabial differences have been well-reported in the adult population for use in the planning of esthetic rhinoplasty and have been shown to vary within each group.^4,6,8,9^ This variability highlights the importance of understanding the specific morphological differences caused by the cleft lip to design effective surgical interventions. While the Hispanic nasal form was initially differentiated by geographic origin,^17–19^ it was later determined that variability existed in all regions and was thus separated instead by deformity type.^20^ Hispanic patients in our control group most closely resemble the described type II form, also called the Mexican American type nose, which is a low radix, normal dorsum, and dependent tip and most closely resembles the proportions of the Caucasian population.^20^ Previous literature on the nasolabial features of African American adults commonly reports thick sebaceous skin, wide alar bases with alar flaring, wide dorsum, bulbous tip, short nasal length, and low radix.^5,7,21–24^ These features are consistent with the anthropometric findings in our African American control group.

In UCLP patients, the nasal deformity involves an asymmetric nose with a flattened, wide retro-displaced nostril on the cleft side and deviated columella and caudal septum towards the non-cleft side.^3^ In our study, Caucasian and Hispanic patients demonstrated a wide nostril on the cleft side through increased alar and alar base widths. All three racial groups with UCLP had an acute nasolabial angle and a wider columella, possibly due to primary rhinoplasty at the time of cleft lip repair. The tip widths were also wider, likely due to the posterolateral displacement of the lower lateral cartilage dome and an increased angle between the medial and lateral crura on the cleft side, which causes the tip to appear amorphous and difficult to landmark. The major difference between UCLP groups was found in the alar and alar base widths. This finding correlated with the pattern in control patients, with Caucasians having the smallest width, followed by Hispanics and African Americans. These differences in nasal and columella heights and widths are important when planning surgery to achieve symmetry. The nasal and columella heights were shorter for African Americans than for Caucasian and Hispanic patients. This should be considered when adjusting tip projection.

The BCLP secondary nasal deformity is described as having a symmetrical nose with inferiorly and posteriorly displaced wide and flattened nostrils bilaterally, a flat, broad nasal tip, and a short columella.^3^ These characteristic findings were shown in our measurements by short and wide nostrils, wide nasal tip, and increased nasolabial angle in all three groups. Additionally, the upper lip and philtrum length were shorter in all groups. In all three group, the nasolabial angle showed wide variation. Bearn et al. stated that while the nasolabial angle remains a commonly used measure for surgical outcomes wide variation is often present at baseline.^25^ The variation may be due in part to the differences in primary rhinoplasty interventions performed at time of cleft lip repair. Caucasian and Hispanic patients also showed wide alar and alar base widths consistent with classical characteristics. When comparing BCLP groups, a pattern of increasing width of the alar and alar base was shown from Caucasians to Hispanics to African Americans. Like the UCLP patients, racial and ethnic variability should be considered when surgically adjusting the alar base's width. Notably, the African American BCLP group's alar base width was not significantly different from the control group. The low number of patients in the UCLP, BCLP, and control African American groups is a limitation of this study. It is important to further investigate and establish a baseline of nasolabial data to inform surgical planning for this patient population.

A thorough understanding in the variation in nasolabial anastomy among different racial and ethnic groups is critical in ensuring the best surgical outcomes. At the same time, it is equally important to consider the patient's esthetic goals and desires. A personalized and comprehensive treatment approach should balance the standard racial and ethnic appearance with the patient's esthetic aspirations, creating a result that is both esthetically pleasing and anatomically sound. By considering both factors, surgeons can provide the best care for their patients and ensure successful surgical outcomes.

## Conclusions

This study provides a comprehensive nasolabial analysis in patients with cleft nasal deformity at dentoskeletal maturity, before secondary rhinoplasty, across racial and ethnic groups. The results of this study can inform surgical planning for secondary cleft rhinoplasty to achieve esthetically pleasing outcomes. The findings demonstrate significant differences in nasolabial anthropometry among racial and ethnic groups, both in control patients and in patients with UCLP or BCLP. These differences should be considered when planning surgical interventions to correct cleft nasal deformities. Further research is needed to fully understand the implications of these differences for surgical planning and to improve outcomes for patients with cleft lip and palate.
